# Effects of Prosocial and Hope-Promoting Communication Strategies on COVID-19 Worry and Intentions for Risk-Reducing Behaviors and Vaccination: Experimental Study

**DOI:** 10.2196/41959

**Published:** 2023-08-01

**Authors:** Elizabeth Scharnetzki, Leo Waterston, Aaron M Scherer, Alistair Thorpe, Angela Fagerlin, Paul K J Han

**Affiliations:** 1 Center for Interdisciplinary Population and Health Research MaineHealth Institute for Research Portland, ME United States; 2 Department of Internal Medicine University of Iowa Iowa City, IA United States; 3 Department of Population Health Sciences University of Utah School of Medicine Salt Lake City, UT United States; 4 Salt Lake City VA Informatics Decision-Enhancement and Analytic Sciences Center for Innovation University of Utah School of Medicine Salk Lake City, UT United States; 5 Tufts University School of Medicine Boston, MA United States; 6 Division of Cancer Control and Population Sciences National Cancer Institute Bethesda, MD United States

**Keywords:** COVID-19, communication, hope, prosocial, vaccination, risk, behavior, vaccine, effect, communication, effectiveness, social, messages, public, web-based, survey

## Abstract

**Background:**

The COVID-19 pandemic has engendered widespread fear and skepticism about recommended risk-reducing behaviors including vaccination. Health agencies are faced with the need to communicate to the public in ways that both provide reassurance and promote risk-reducing behaviors. Communication strategies that promote prosocial (PS) values and hope are being widely used; however, the existing research on the persuasiveness of these strategies has offered mixed evidence. There is also very little research examining the comparative effectiveness of PS and hope-promoting (HP) strategies.

**Objective:**

The aim of this study is to evaluate the comparative effectiveness of PS and HP messages in reassuring the public and motivating COVID-19 risk–reducing behaviors.

**Methods:**

A web-based factorial experiment was conducted in which a diverse sample of the US public was randomized to read messages which adapted existing COVID-19 information from a public website produced by a state government public health department to include alternative framing language: PS, HP, or no additional framing (control). Participants then completed surveys measuring COVID-19 worry and intentions for COVID-19 risk–reducing behaviors and vaccination.

**Results:**

COVID-19 worry was unexpectedly higher in the HP than in the control and PS conditions. Intentions for COVID-19 risk–reducing behaviors did not differ between groups; however, intentions for COVID-19 vaccination were higher in the HP than in the control condition, and this effect was mediated by COVID-19 worry.

**Conclusions:**

It appears that HP communication strategies may be more effective than PS strategies in motivating risk-reducing behaviors in some contexts but with the paradoxical cost of promoting worry.

## Introduction

The COVID-19 pandemic has been one of the worst public health crises of modern history not only due to the physical effects of the disease itself but due to its psychological and social effects on members of the general public [1,2]. These effects include widespread fear and anxiety, as well as distrust of health information and experts; antisocial attitudes and behaviors including scapegoating, stereotyping, discrimination, and spread of misinformation; and skeptical attitudes toward recommended risk-reducing behaviors including vaccination [3-7]. These negative psychological and social responses have posed great challenges for efforts to control the COVID-19 pandemic. They have raised the need to communicate to the public in ways that both provide reassurance and promote risk-reducing behaviors—2 tasks identified in the Centers for Disease Control and Prevention’s Crisis and Emergency Risk Communication guidelines as key management goals of all public health crises [8].

The unanswered question, however, is how best to achieve these goals. To date, COVID-19 information campaigns have used various strategies. One dominant strategy, used in numerous campaigns, has been to appeal to prosocial (PS) or communal values that appeal to concern for the common good (eg, “We’re in this together–and we will get through this, together” [9]). PS messages are thought to heighten positive emotions that may increase confidence in COVID-19 vaccines and diminish negative emotions that may have the opposite effects [10,11]. Although direct evidence on the effectiveness of PS appeals on COVID-19 vaccination or other preventive behaviors is limited [12], indirect evidence from studies of basic social-psychological processes and of the effects of PS messaging strategies on other preventive health behaviors provide support for this recommendation [13].

Research on PS motivation has further shown that behavioral messages that focus attention on others (“others-focused framing”) can be perceived as more persuasive than messages that focus attention on oneself (“self-focused framing”) [14,15]. Luttrell and Petty [14] have shown that people perceive “others-focused messaging” about social distancing as more persuasive than self-focused messaging and posited that this is because others-focused messages serve as moral arguments for adopting behaviors [10]. For example, health professionals were more likely to comply with hand hygiene recommendations when presented with messages that focused on patient safety as opposed to their own safety [16]. In the context of the COVID-19 pandemic, however, empirical research has yielded conflicting results. While Jordan et al [17] provided evidence to suggest that other-focused (vs self-focused) message framing strategies were strongly associated with increased intentions to engage in risk-reducing behaviors, Banker and Park [18] found that some PS message frames (eg, “protect your community”) were less effective than self-focused frames in motivating the general public to engage with COVID-19 information on prevention [14]. This work contributes to a large body of social-psychological research focused on examining whether people are motivated to conform to social norms, particularly in situations of uncertainty [10,19].

Nevertheless, the effectiveness of PS appeals in promoting COVID-19–preventive behaviors is unknown, and existing evidence is mixed. In a social media experiment using Facebook, community-focused PS messages were shown to be less effective than self-focused messages in generating engagement and interest in obtaining further information on COVID-19 [18]. Similar results were obtained in an Associated Press National Opinion Research Center public opinion survey, in which the desire to protect one’s self, as opposed to one’s family or community, was the most frequently cited motivation for getting vaccinated [20].

Another widely used strategy to reassure the public and promote COVID-19 risk–reducing behaviors has focused on reinforcing positive future expectancies, which encompass hope—that is, positive expectations regarding future outcomes and one’s own ability to achieve them [21]—and the broader construct of optimism—that is, generalized expectations of positive outcomes [22]. Like fear, hope is thought to be a future-focused emotion [23]. Hope anchors one’s cognitions toward either obtaining future rewards or avoiding punishment. These outcomes are known to be strong predictors of behavior; thus, hope is theoretically a persuasive communication tool [24]. Furthermore, the theoretical effectiveness of messages that evoke hope is supported by empirical evidence showing an association between the related construct of optimism and physical and mental well-being [22,25-32]. Drawing on appraisal theories of emotion in the psychological literature, Chadwick [23] defined hope as an emotion evoked by appraisals of a future outcome as being: (1) consistent with goals (goal congruence), (2) possible but not certain (possibility), and (3) important (importance), and leading to a better future (future expectation). Chadwick [23] further defined hope appeals as messages that evoke these appraisals in order to persuade people to take advantage of a given opportunity.

The theoretical effectiveness of hope-promoting (HP) messages in motivating COVID-19 risk–reducing behaviors is also supported by both theory and empirical evidence on the mediating role of positive emotions in promoting such behaviors [10,11,28,33]. HP messages may leverage individuals’ inherent “optimism bias”—the tendency to believe that bad things are less likely to happen to oneself than others [34]. This bias serves the adaptive functions of helping people moderate feelings of uncertainty and anxiety and decreasing defensive responses to threats [35]. It also prevents excessively pessimistic appraisals and negative emotions that can diminish motivations to take action.

Empirical researches examining the effects of health messages that evoke hope (hope appeals) have been limited and have yielded somewhat inconsistent results. In 1 study, climate change messages designed to evoke hope were perceived as effective and interesting but did not significantly affect participants’ intentions to engage in climate-protecting behaviors [23]. These findings suggest that hope appeals by themselves may not be persuasive enough to motivate behavior, although it remains to be seen whether such appeals might be effective if combined with specific recommendations for action [23]. Another study found that hopefulness and self-efficacy may promote changes in behavioral intentions [36]. Specifically, this study demonstrated that informational messages about sun safety were most effective in promoting intentions to engage in sun protection among participants who were high in both hope and self-efficacy [36].

HP messages have been commonly employed in COVID-19 communication campaigns (eg, “Spread Hope, Not COVID” [37]); however, the effects of HP messages on emotions and behaviors related to COVID-19 have not been empirically evaluated, and their effects may not always be positive. For example, HP messages might promote excessively optimistic appraisals that diminish motivations for protective behaviors [10,34], leading people to discount information about the risks of COVID-19 and the benefits of preventive behavior [10]. On the contrary, health messages may have positive effects on preventive behaviors such as vaccination, but these effects may be mediated by negative effects on emotions, such as heightened worry about health risks [38].

Behavioral theories and empirical research studies thus provide some support for the use of PS and HP communication strategies as a means of reassuring the public and promoting risk-reducing behaviors during public health crises [23,39]. To our knowledge, however, direct empirical evidence for both the absolute and relative effectiveness of these strategies in actual crises, such as the current COVID-19 pandemic, is lacking. This is an important knowledge gap, given the widespread use of PS and HP messages in existing public information campaigns. To effectively and efficiently manage the COVID-19 crisis, public health officials and other key stakeholders need to understand the effects of these messages.

The objective of this study was to test the efficacy of messages that use PS and HP strategies in encouraging COVID-19 risk–reducing behaviors. While there have been numerous messaging strategies used during the COVID-19 pandemic, the existing theoretical frameworks underlying these strategies make evaluating the comparative effectiveness of PS and HP messages both pragmatic and scientifically interesting. We aimed to test whether the PS and HP messages would be effective in (1) reassuring the public (operationalized in this study by decreasing worry about COVID-19) and (2) motivating risk-reducing behaviors (operationalized in this study by increasing intentions to adhere to COVID-19 risk–reducing behaviors and vaccination). With this question in mind, we had 2 primary hypotheses. First, we hypothesized that compared to neutral messages that simply recommended COVID-19 risk–reducing behaviors and vaccination, both PS and HP messages would decrease COVID-19 worry (ie, provide reassurance). Second, we hypothesized that compared to neutral messages that simply recommended COVID-19 risk–reducing behaviors and vaccination, both PS and HP messages would increase intentions for COVID-19 risk–reducing behaviors. Assessing the comparative effectiveness of PS and HP messages was a central aim of this study. We did not have a directional hypothesis for the superiority of either strategy given the lack of theoretical or empirical evidence. We also had several exploratory aims, including the extent to which the effects of PS and HP messages were moderated and mediated by various factors. In previous investigations, sociodemographic factors including age and political affiliation have been shown to affect responses to COVID-19 health messages [40]. In this study, we, therefore, examined whether age and political affiliation would moderate the effects of HP and PS messages on participants’ worry about COVID-19 and intentions to engage in risk-reducing behaviors and vaccination. As a secondary exploratory aim, we also assessed whether COVID-19 worry might mediate the relationship between messaging strategies and vaccination intentions, based on previous research suggesting a mediating role of worry in the effect of different hypothetical influenza messages on vaccination intentions [38].

## Methods

### Study Design and Experimental Manipulation

This study was part of a larger digital survey–based factorial experiment designed to test the effects of alternative strategies for communicating about the COVID-19 pandemic, including strategies for communicating about scientific uncertainty [40]. The experiment was conducted early in the trajectory of the COVID-19 pandemic (May 7 to June 11, 2020), and this study focused on comparing the effects of PS and HP messages.

We developed the experimental messages by adapting basic COVID-19 information from a public website produced by a state government public health department. The information discussed the nature of the COVID-19 pandemic and the need for risk-reducing behaviors and promoted multiple behaviors at once (eg, social distancing, sheltering in place, and mask use). This information served as the control strategy (C), which we then supplemented with either PS or HP language to produce a total of 3 alternative communication strategies to which study participants were randomly assigned: (1) control, (2) PS, and (3) HP (Multimedia Appendix 1). We chose to retain the control message’s focus on multiple risk-reducing behaviors in all 3 conditions in order to maximize the ecological validity of our findings, although messages focusing on a particular aspect of the problem or a single risk-reducing behavior may have been more persuasive [41]. The PS condition contained additional language encouraging concern for the collective good, for example,

To protect our country from the COVID-19 pandemic, we all need to put aside our differences and join together. It is important that we not only protect ourselves, but also one another, including our families, loved ones, neighbors, and our communities. Our health is one of the most important things we have in life, and by working together, we can preserve our health and make our world a safer place. We’re all in this together.

We designed the HP condition to convey more positive general expectations about future control over the pandemic [42], for example,

Lock-downs, strict sheltering-in-place regulations, and social distancing practices have been successful in controlling the spread of the coronavirus, and fewer people are becoming infected with COVID-19 and dying from it. These are very encouraging signs that our hard work is paying off, and we are gaining control of this problem and making it through the crisis.

The message thus promoted the key appraisal dimensions deemed essential for hope, in emphasizing that control of the COVID-19 pandemic was possible, important, consistent with people’s goals, and leading to a better future [23].

### Study Population and Recruitment

The study population consisted of a national sample of 915 members of the general public who belong to voluntary opt in web survey panels professionally managed by the internet survey vendor Qualtrics and who receive monetary compensation for their survey panel participation. Qualtrics maintains panel members’ sociodemographic and geographic data, which enables the recruitment of a diverse study sample using prespecified quotas. This study established quotas aimed to achieve a balanced distribution of age, gender, education (≥20% high school diploma or less), and income (≥50% annual income of US $50,000 or less). The survey vendor used established screening protocols to exclude participants who gave low-effort responses; these included “straightlining” responses and total survey completion time of less than 12 minutes, given the length of the questionnaire. We further excluded 14 participants who reported being currently or previously diagnosed with COVID-19, given the potential for personal disease experience to influence COVID-19 risk perceptions [42].

### Ethics Approval

The study was deemed exempt and approved by the MaineHealth institutional review board (1584932). Participants were provided with a study information sheet and provided implied consent by choosing to participate. The survey collected sociodemographic data (age, gender identity, and income), but no personal identifiable information were included. Participants were Qualtrics panel members who receive monetary compensation for completing surveys. The study team worked with Qualtrics project managers to ensure respondents received modest monetary compensation for their participation, which was provided and managed by the survey vendor.

### Measures

#### Overview

After reading the informational messages, participants completed several survey measures.

#### Outcome Variables

##### Worry About COVID-19

Worry about COVID-19 was measured with a single item used in prior studies [40,43]: “How worried are you about getting COVID-19 within the next month.” A 7-point Likert scale was used, with response options ranging from 1 (not at all) to 7 (very).

##### Intentions for COVID-19 Risk–Reducing Behaviors

Intentions for COVID-19 risk–reducing behaviors were measured with a series of questions used in a prior study [43] and assessing participants’ ratings of their willingness to adhere to 14 Centers for Disease Control and Prevention–recommended COVID-19 risk–reducing guidelines (Multimedia Appendix 1)*.* Likert scale response options ranged from 0 (I am not planning to follow this guideline at all) to 100 (I am planning to follow this guideline fully). Participants’ responses were averaged to create a composite score (=.95).

##### Intentions for COVID-19 Vaccination

Intentions for COVID-19 vaccination were measured with a single item: “If a vaccine becomes available for COVID-19, how likely would you be to get vaccinated against COVID-19?” A 7-point Likert scale was used, with response options ranging from 1 (Definitely would not get a vaccination) to 7 (Definitely would get a vaccination).

### Potential Moderators

Sociodemographic characteristics included age (grouped in the following categories used to recruit the sample: <30, 30-39, 40-49, 50-59, 60-69, and ≥70 years), gender, race, and political affiliation (Democrat, Independent or other, Republican).

### Data Analysis

To assess the effects of our communication strategies on our outcome measures, we fit univariate ANOVA models with risk perceptions, worry, guideline adherence, and intentions to vaccinate specified as the dependent variables, and communication strategy condition specified as the independent variable. For each dependent variable, we evaluated prespecified contrasts between (1) the control condition and the HP condition; (2) the control condition and the prosocial motivation condition; and (3) the HP and PS conditions to compare the effects of each strategy relative to one another.

To explore the potential moderating effects of sociodemographic characteristics (age and political affiliation), we fit separate ANOVA models including relevant interaction terms. Communal orientation composite scores were dichotomized by a median split into high and low categories because of a skewed distribution of responses. All analyses were conducted using SPSS statistical software (version 27; IBM Corp). We conducted exploratory mediational analyses, using the SPSS macro PROCESS (Model 4, with multicategorical predictors specified [44]), to evaluate the extent to which different messaging strategies influenced vaccination intentions indirectly, through their effects on worry [38].

## Results

### Overview

We received data on a total of 915 respondents randomized to the experimental conditions tested in this study. We excluded 14 individuals who reported current or previous COVID-19 illness, leaving a final sample of 901 respondents (Table 1). Data were assumed to be missing at random; thus, we used a listwise deletion strategy for participants with missing data on any outcome measures.

On average, participants took 28.70 (SD 34.85) minutes to complete the study. No significant differences in the time of completion of the experimental task were observed between conditions (*F*_2,898_=0.25; *P*=.78), suggesting that the cognitive effort required by the task was similar across conditions.

**Table 1:** Sample characteristics.^a^

Characteristics			Values, n (%)
**Age (years)**			
	<30	193 (21.4)	
	30-39	124 (13.8)	
	40-49	110 (12.2)	
	50-59	121 (13.4)	
	60-69	156 (17.3)	
	≥70	197 (21.9)	
**Gender**			
	Male	400 (44.4)	
	Female	500 (55.5)	
**Race**			
	White	576 (63.9)	
	Black or African American	120 (13.3)	
	Asian (including Pacific Islander)	104 (11.5)	
	Hispanic or Latino	43 (5)	
	Multiracial or other	58 (6)	
**Education**			
	Less than high school	163 (18.1)	
	High school graduate	144 (16)	
	Some college or trade school	249 (27.6)	
	College graduate or higher	345 (38.2)	
**Income**			
	US $0- US $24,999	246 (27.4)	
	US $25,000-US $49,999	229 (25.4)	
	US $50,000-US $99,999	218 (24.2)	
	US $100,000-US $149,999	135 (15)	
	≥US $150,000	73 (8)	
**Political affiliation**			
	Democrat	335 (37.2)	
	Republican	259 (28.7)	
	Independent	180 (20)	
	Other	127 (14.1)	

^a^Not all numbers add to 901 due to missing data.

### Worry About COVID-19

There was a significant main effect of experimental condition on worry about COVID-19 (*F*_2,898_=6.01; *P*=.003; η^2^=0.013); however, contrary to hypotheses, the level of worry was higher, rather than lower, in the HP condition relative to both the control (*P*=.002; *d*=–0.26) and the PS conditions (*P*=.005; *d*=0.23). Worry was not significantly different in the PS condition compared to that in the control condition (*P*=.757; *d*=–0.03).

### Intentions for COVID-19 Risk–Reducing Behaviors

Inconsistent with hypotheses, there was no significant main effect of either the PS or HP messages on intentions regarding COVID-19 risk–reducing behaviors, (*P*=.18; η^2^=0.004).

### Intentions for COVID-19 Vaccination

Consistent with hypotheses, there was a significant main effect of experimental condition (*F*_2,898_=3.80; *P*=.02; η^2^=0.01) on COVID-19 vaccination intentions. Prespecified contrasts demonstrated that vaccination intentions were significantly higher for participants in the HP condition relative to participants in the control condition (*P*=.007; *d*=–0.22); there was also a trend toward higher vaccination intentions in the HP compared to the PS condition (*P*=.07; *d*=0.15). Vaccination intentions did not significantly differ between the PS and the control conditions (*P*=.39; *d*=–0.07).

### Moderating Effects

**Figure 1:**
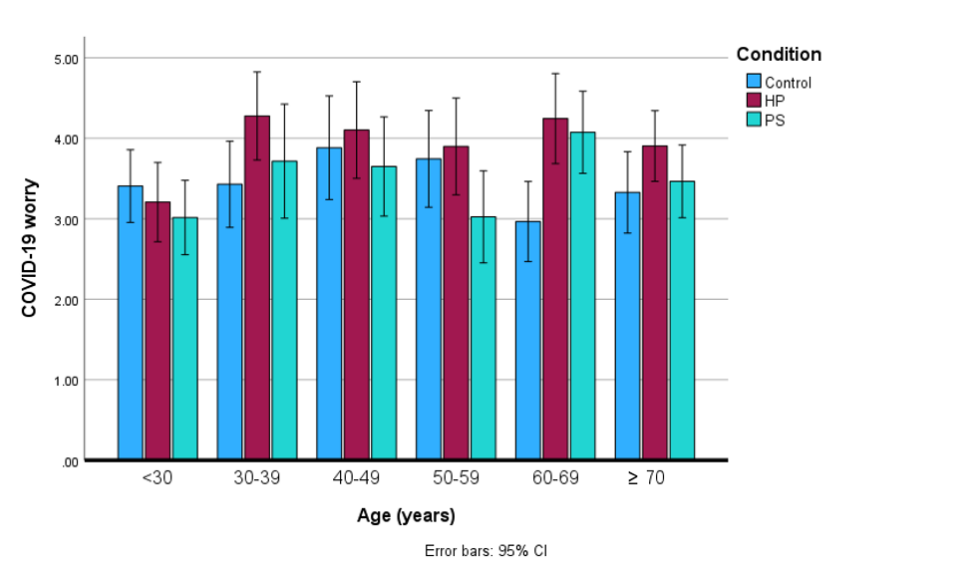
Moderating influence of age on the effects of messaging strategy on COVID-19 worry. HP: hope promoting; PS: prosocial.

Only 1 sociodemographic factor, age, was found to moderate the effects of HP and PS messages on participants’ worry about COVID-19 (*F*_5,883_=1.82; *P*=.05; ηρ²=0.02); younger participants (aged 30 and younger) in the HP and PS conditions generally reported being less worried about COVID-19 compared to the control condition (Figure 1). There were no significant interactions between any of the other potential moderators and communication messages on any of the outcome variables.

### Mediating Effects

**Figure 2:**
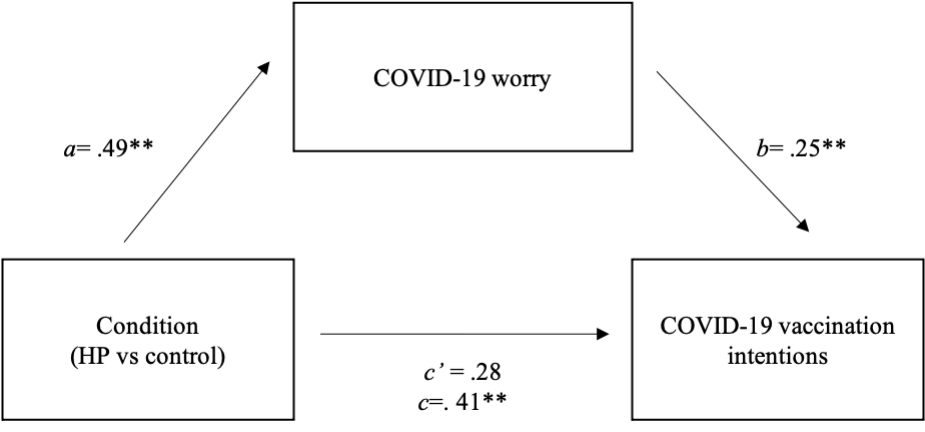
Effects of the hope-promoting communication strategy on COVID-19 worry and vaccination intentions. a, b, and c represent unstandardized regression coefficients for the associations between condition (HP vs control), worry, and COVID-19 vaccination intentions; c’ represents the unstandardized regression coefficient for the association between condition (HP vs control) and COVID-19 vaccination intentions, adjusting for COVID-19 worry.*P≤05; **P≤01. HP: hope promoting.

Given that significant associations between communication strategy and both COVID-19 worry and vaccination intentions were present only for the HP message, we restricted our exploratory mediational analysis to this set of relationships. Supporting the mediational hypothesis, the HP strategy (compared to control) was significantly associated with COVID-19 vaccination intentions (*b*=0.41; *P*=.007). The HP condition was also significantly associated with greater COVID-19 worry (*b*=0.49; *P*=.002), and greater worry was associated with greater vaccination intentions (*b*=0.25; *P*<.001). Consistent with full mediation, the effect of HP strategy (relative to control) on intentions to vaccinate was significantly reduced after controlling for COVID-19 worry (*b*=0.28; *P*=.05). The indirect effect, estimated using a percentile bootstrap estimation approach with 5000 samples [44], was significant (*b*=0.125; SE 0.04; 95% CI 0.04-0.22). A Sobel test confirmed that the association between HP condition and COVID-19 vaccination intentions was fully mediated by COVID-19 worry (Sobel *z*=2.94; *P*=.003; Figure 2).

## Discussion

This study evaluated the comparative effectiveness of PS and HP communication strategies in decreasing worry about COVID-19 and increasing intentions for COVID-19 risk–reducing behaviors and vaccination among the US public. Although these strategies have been commonly used by public health professionals and agencies to encourage COVID-19 preventive behaviors among the general public, there has been little empirical examination of their effectiveness. We believe our study provides several important insights for future efforts to communicate information about COVID-19 and other public health threats.

First, our study showed that neither a PS nor an HP communication strategy was effective in increasing intentions for general COVID-19 risk–reducing behaviors. Other studies have shown similarly limited effects of such strategies [43], and these findings may be due to several factors. The first is the historical context. At the time the study was conducted, many of the risk-reducing behaviors assessed in this study were legally mandated (eg, wearing a mask), and COVID-19 cases and deaths were rapidly increasing. These factors may have lessened the impact of health messages on public interest in COVID-19 risk–reducing behaviors.

Importantly, however, our study did show that an HP strategy was effective in increasing intentions for COVID-19 vaccination, while a PS strategy had no effect. To our knowledge, this finding has not been previously reported and may provide at least some empirical support for the use of HP communication strategies. However, this finding also needs to be temporally contextualized. At the time the study was conducted, early in the course of the pandemic, COVID-19 vaccines had been developed, and vaccine hesitancy was thus not a topic of public discourse. In the current historical context, in which multiple COVID-19 vaccines are available and vaccination has become intensely debated and politicized, the effectiveness of HP, PS, and other communication strategies may have different effects. Furthermore, participants may already have encountered COVID-19 messages similar to those used in our control and experimental conditions, and familiarity with these messages—which we did not assess—could have affected the results. Our study needs to be repeated to determine the extent to which historical factors moderate the effects of different communication strategies.

Our study also yielded an unexpected and potentially important finding regarding participants’ worry about COVID-19: The HP communication strategy increased worry, rather than decrease it. This finding is inconsistent with the hypothesis—reflected in prevailing expert opinion and COVID-19 communication efforts—that HP messages promote positive emotions, engagement, and reassurance [11]. More research is needed to determine the reasons for the paradoxical worry-promoting effect of the HP message in our study; however, ironic process theory may offer 1 explanation. Deliberate attempts to suppress thoughts or feelings (such as fear and worry that COVID-19 elicits in the general public) may cognitively “activate” and ultimately bringing these cognitions and emotions to the “surface” [45]. Ironic activation occurs through dual cognitive processes: (1) individuals unconsciously monitor and steer their cognitions away from undesirable thoughts or feelings (operating process) and (2) if detected (or primed), these cognitions enter conscious awareness (monitoring process) [45]. In the case of the COVID-19 pandemic, HP messages aimed at reassurance may thus paradoxically activate worry or increase individuals’ attention to it.

Yet, our study also showed that the elevated worry caused by the HP strategy had the beneficial effect of mediating the positive influence of the HP message on COVID-19 vaccination intentions. To our knowledge, this finding has not been previously reported and needs to be replicated, and we can only speculate on its mechanisms and implications. If real, the mediating influence of worry suggests a paradox: HP messages may increase individuals’ vaccination intentions not by providing reassurance but by exacerbating worry about COVID-19. It is possible that evoking appraisals of a future outcome as goal-congruent, possible, important, and desirable (key appraisals for hope) may also increase people’s anxiety about the possibility of not realizing the outcome [23]. This finding suggests a potential trade-off in communication efforts: HP messages may promote vaccination intentions but at the cost of potentially diminished emotional well-being. Health communicators who use this strategy may need to consider this potential trade-off and enact measures to mitigate the potential worry-inducing effects of promoting hope.

This study has several limitations that qualify the interpretation of our results. The study sample, while large and relatively sociodemographically diverse, consisted of Qualtrics panel members who are experienced in and receive monetary compensation for completing surveys. Social desirability response bias is thus a possible limitation in this study, and more research is needed to replicate our findings in other more diverse sample populations. We also did not include attention checks in this study; given the length of our informational messages and surveys, we cannot rule out survey fatigue as a potential source of error in our findings. Additionally, we did not pretest the experimental messages nor did we conduct experimental manipulation or attention checks. Although such checks introduce methodological problems of their own, more work is needed to evaluate the effectiveness of our experimental messages [46]. Importantly, our key outcome measures consisted only of intentions for COVID-19 risk–reducing behaviors and vaccination, not actual behaviors. Although intentions are critical determinants and reasonable proxies for behaviors [47], further research is needed to investigate the effects of HP messages on actual risk-reducing behaviors and vaccination uptake. Two of our outcome measures (COVID-19 worry and vaccination intentions) used single items. More work is needed to assess the reliability of these measures and to replicate our findings using alternative measures. We also did not test the joint effects of PS and HP messages with one another; whether this or other combinations of messaging strategies are beneficial is an important question for future research. Additionally, we did not assess respondents’ motivation to process different messages or the extent to which they engaged in deliberative processing; these are other important topics for future research. Furthermore, our HP messages were designed to promote positive general expectations about the future; however, researchers have often conceptualized and measured this phenomenon using the construct of optimism and used hope to refer to a broader phenomenon encompassing both positive expectancies and beliefs about one’s ability to achieve them [21,48-50]. Future research should assess whether alternative messages designed to promote “hope”—conceptualized and measured in this or other ways—have similar effects. Similarly, PS messages can be difficult to evaluate and may elicit a social desirability bias from respondents. Future research should include measures to assess and control the impact of social desirability on people’s responses to different messaging strategies. Finally, we analyzed several associations and did not correct the multiple comparisons given the exploratory nature of our study; some of our findings may thus be attributable to change and will need to be replicated in future studies.

Despite these limitations, we believe our study provides valuable empirical evidence on the effectiveness of HP and PS communication strategies on the public’s psychological and behavioral responses to information about COVID-19. HP strategies may be effective in promoting vaccination intentions, in at least some circumstances. More research is needed to confirm and better understand the effects of these and other communication strategies, and the generalizability of these effects to other public health crises.

## Appendix

Multimedia Appendix 1 Experimental messages.

## Abbreviations

HP: hope promoting
PS: prosocial

## Data Availability

The data sets used and analyzed during this study are not publicly available due to institutional review board compliance restrictions. Syntax available upon reasonable request from the corresponding author.
