# Supplementary material for: Effects of Prosocial and Hope-Promoting Communication Strategies on COVID-19 Worry and Intentions for Risk-Reducing Behaviors and Vaccination: Experimental Study
Source: JMIR Form Res. 2023 Aug 1;7:e41959. doi: 10.2196/41959 (PMC10411423; doi:10.2196/41959)
Supplement: Multimedia Appendix 1 [file formative_v7i1e41959_app1.docx]

**Control Condition Message:**

**2019 Novel Coronavirus (COVID-19)**
This survey is about the 2019 Novel Coronavirus (COVID-19) outbreak, or pandemic—a public health crisis that has affected the whole world and every state in the US.

The survey begins with some information about COVID-19.  Please take your time and read this information carefully.  The survey will then ask you about your attitudes and opinions regarding the COVID-19 pandemic.

**Spread and Symptoms of COVID-19**
COVID-19 is a respiratory illness caused by a coronavirus, a type of virus that infects human beings.  It is extremely contagious and spreads from person to person through contact with respiratory secretions from an infected person (e.g., through sneezing and coughing).  Anybody can get COVID-19, and nobody is risk-free.  
 
Illness caused by COVID-19 can range from mild to severe.  Some people can be infected and have no symptoms at all.  More commonly, people with COVID-19 have cough and difficulty breathing, or at least two of these other symptoms:  fever, chills, repeated shaking with chills, muscle pain, headache, sore throat, or new loss of taste or smell.  Some people have serious illness which can include pneumonia, kidney failure, and death.  People who are older and who have other medical problems are at higher risk of dying from COVID-19.
 
**Prevention and Treatment of COVID-19**
Currently, there is no vaccine that can prevent COVID-19 infection.  The only way to prevent COVID-19 and to control the pandemic is to keep it from spreading.  Current recommendations include the following:

- Practice good hygiene, including handwashing and use of hand sanitizers
- Practice social distancing (putting space between you and other people)
- Wear a mask
- Self-isolation and self-quarantine:  stay home and avoid contact with other people if you’re sick with COVID-19, or have symptoms of it (Self-isolation), or if you may have been exposed to someone with COVID-19 (Self-quarantine)

In addition to these measures, state and local governments in the US have enacted strict sheltering-in-place regulations, travel bans, and “lock-downs” of businesses and schools, in order to limit the spread of the coronavirus.  
 
Currently, there is no effective, widely available treatment that can cure COVID-19 infection.  Most people recover at home, without treatment.  Some people need to be hospitalized, and require oxygen or breathing machines (mechanical ventilation) to help them recover.  But there is no cure for COVID-19 and some people, especially those who are older and who have other medical problems, do not recover.

**Future Management of the COVID-19 Pandemic**

The COVID-19 pandemic is now starting to slow down in several US states and around the world.  Lock-downs, strict sheltering-in-place regulations, and social distancing practices have been successful in controlling the spread of the coronavirus, and fewer people are becoming infected with COVID-19 and dying from it.  Because of this, several state and city governments are starting to ease or remove these strict measures, and to let people go back to work. 

However, there is a risk that the COVID-19 pandemic could get worse again, and cause more suffering and death.  Because there still is no vaccine or cure, COVID-19 remains a serious threat that can reappear at any time or place.  It can begin spreading again if we stop being careful.  Easing lock-downs, opening up businesses and schools, and relaxing social distancing orders could allow the COVID-19 pandemic to get out of control once again, and put people’s lives at risk.

For now, lock-downs and strict social distancing measures remain in place in many US states.  Many experts believe that several things are necessary before lock-downs and strict social distancing measures are loosened, including the capability to test many more people for the coronavirus, to isolate people who are infected, and to identify and monitor their close contacts.  Many other government officials believe it is safe to begin loosening lock-downs and strict social distancing measures right now.  Some state and local governments in the US have therefore started loosening these measures, or are at least considering loosening doing so. 

**Hope-Promoting Condition:**

**2019 Novel Coronavirus (COVID-19)**
This survey is about the 2019 Novel Coronavirus (COVID-19) outbreak, or pandemic—a public health crisis that has affected the whole world and every state in the US.  We are learning more about it every day. 
  
The survey begins with some information about COVID-19.  Please take your time and read this information carefully.  The survey will then ask you about your attitudes and opinions regarding the COVID-19 pandemic.

**Spread and Symptoms of COVID-19**
COVID-19 is a respiratory illness caused by a coronavirus, a type of virus that infects human beings.  It is extremely contagious and spreads from person to person through contact with respiratory secretions from an infected person (e.g., through sneezing and coughing).  Anybody can get COVID-19, and nobody is risk-free.  Staying alert and watchful will help your reduce your risk of becoming infected and spreading COVID-19 to others.
 
Illness caused by COVID-19 can range from mild to severe.  Some people can be infected and have no symptoms at all. More commonly, people with COVID-19 have cough and difficulty breathing, or at least two of these other symptoms:  fever, chills, repeated shaking with chills, muscle pain, headache, sore throat, or new loss of taste or smell.  Some people have serious illness which can include pneumonia, kidney failure, and death.  People who are older and who have other medical problems are at higher risk of dying from COVID-19.  Knowing and looking out for the symptoms will protect yourself and the people around you. 
 
**Prevention and Treatment of COVID-19**
Currently, there is no vaccine that can prevent COVID-19 infection.  However, scientists are working on developing and testing different possible vaccines, and are optimistic that an effective vaccine will be available some time in the next year.  In the meantime, the only way to prevent COVID-19 and to control the pandemic is to keep it from spreading, and there are many things we can do.  Current recommendations include the following:

- Practice good hygiene, including handwashing and use of hand sanitizers
- Practice social distancing (putting space between you and other people)
- Wear a mask
- Self-isolation and self-quarantine:  stay home and avoid contact with other people if you’re sick with COVID-19, or have symptoms of it (Self-isolation), or if you may have been exposed to someone with COVID-19 (Self-quarantine)

In addition to these measures, state and local governments in the US have enacted strict sheltering-in-place regulations, travel bans, and “lock-downs” of businesses and schools, in order to limit the spread of the coronavirus.  Taking care and doing all you can to follow these recommendations will keep you and everyone around you safer. 
 
Currently, there is no effective, widely available treatment that can cure COVID-19 infection.  However, the research is promising, and scientists are hopeful that they will find an effective treatment soon.  Most people recover at home, without treatment.  Some people need to be hospitalized, and require oxygen or breathing machines (mechanical ventilation) to help them recover.  But there is no cure for COVID-19 and some people, especially those who are older and who have other medical problems, do not recover.  But good medical care will help many people make it through the infection.
 
 **Future Management of the COVID-19 Pandemic**

The COVID-19 pandemic is now starting to slow down in several US states and around the world.  Lock-downs, strict sheltering-in-place regulations, and social distancing practices have been successful in controlling the spread of the coronavirus, and fewer people are becoming infected with COVID-19 and dying from it.  These are very encouraging signs that our hard work is paying off, and we are gaining control of this problem and making it through the crisis.  Because of this, several state and city governments are starting to ease or remove these strict measures, and to let people go back to work.        

However, there is a risk that the COVID-19 pandemic could get worse again, and cause more suffering and death.  Because there still is no vaccine or cure, COVID-19 remains a serious threat that can reappear at any time or place.  It can begin spreading again if we stop being careful.  Easing lock-downs, opening up businesses and schools, and relaxing social distancing orders could allow the COVID-19 pandemic to get out of control once again, and put people’s lives at risk.

For now, lock-downs and strict social distancing measures remain in place in many US states.  Many experts believe that several things are necessary before lock-downs and strict social distancing measures are loosened, including the capability to test many more people for the coronavirus, to isolate people who are infected, and to identify and monitor their close contacts.  Many other government officials believe it is safe to begin loosening lock-downs and strict social distancing measures right now.  Some state and local governments in the US have therefore started loosening these measures, or are at least considering loosening doing so.  Regardless of what course we choose, however, we need to do our very best to reduce the threat of COVID-19.  This means staying cautious and not letting up but keeping up our efforts to prevent, detect, and treat this deadly disease, until this disease is eliminated.  If we continue to work hard to protect ourselves, we will make it through this crisis, stronger than we were before.
 

**Pro-Social Condition:**

**2019 Novel Coronavirus (COVID-19)**

This survey is about the 2019 Novel Coronavirus (COVID-19) outbreak, or pandemic—a public health crisis that has affected the whole world and every state in the US.    

During this time of crisis in our country and our world, our connections to one another become more important than ever. Relationships with our families, our loved ones, and our communities provide us with courage and support, and it is through our connections with others that we are able to navigate tough situations. Please take a moment and think about what the connections in your life mean to you.
 
We would now like you to take the following survey, which begins with some information about COVID-19.  Please take your time and read this information carefully.  The survey will then ask you about your attitudes and opinions regarding the COVID-19 pandemic.
 
**The COVID-19 Pandemic**
 
To protect our country from the COVID-19 pandemic, we all need to put aside our differences and join together.  It is important that we not only protect ourselves, but also one another, including our families, loved ones, neighbors, and our communities. Our health is one of the most important things we have in life, and by working together, we can preserve our health and make our world a safer place. 
 
We’re all in this together.
 
**Spread and Symptoms of COVID-19**
Knowing how COVID-19 is spread can help you stay healthy, and reduce the risk of infection for you, your loved ones, and other people in your community. COVID-19 is no one’s fault, but everyone’ responsibility.
 
COVID-19 is a respiratory illness caused by a coronavirus, a type of virus that infects human beings.  It is extremely contagious and spreads from person to person through contact with respiratory secretions from an infected person (e.g., through sneezing and coughing).  Anybody can get COVID-19, and nobody is risk-free. That means we all need to prevent spreading the COVID-19 virus to each other. 
 
Illness caused by COVID-19 can range from mild to severe.  Some people can be infected and have no symptoms at all.  More commonly, people with COVID-19 have cough and difficulty breathing, or at least two of these other symptoms:  fever, chills, repeated shaking with chills, muscle pain, headache, sore throat, or new loss of taste or smell.  Some people have serious illness which can include pneumonia, kidney failure, and death.  People who are older and who have other medical problems are at higher risk of dying from COVID-19.  We need to take special care to protect our family members and loved ones who belong to these groups.
 
**Prevention and Treatment of COVID-19**
Currently, there is no vaccine that can prevent COVID-19 infection.  The only way to prevent COVID-19 and to control the pandemic is to keep it from spreading.  Your actions can make the world safer for your loved ones and your community.  Current recommendations include the following:

- Practice good hygiene, including handwashing and use of hand sanitizers
- Practice social distancing (putting space between you and other people)
- Wear a mask
- Self-isolation and self-quarantine:  stay home and avoid contact with other people if you’re sick with COVID-19, or have symptoms of it (Self-isolation), or if you may have been exposed to someone with COVID-19 (Self-quarantine)

Following these recommendations can protect you, your loved ones, and your community.  In addition to these measures, state and local governments in the US have enacted strict sheltering-in-place regulations, travel bans, and “lock-downs” of businesses and schools, in order to limit the spread of the coronavirus.  These measures require us all to share responsibility for controlling COVID-19.  Because it can infect anyone, everyone needs to work together to prevent it. 
 
Currently, there is no effective, widely available treatment that can cure COVID-19 infection.  Most people recover at home, without treatment.  Some people need to be hospitalized, and require oxygen or breathing machines (mechanical ventilation) to help them recover.  But there is no cure for COVID-19 and some people, especially those who are older and who have other medical problems, do not recover.  People infected with COVID-19 should seek medical attention, so that they can receive the best care possible for the illness.

**Future Management of the COVID-19 Pandemic**

The COVID-19 pandemic is now starting to slow down in several US states and around the world.  Lock-downs, strict sheltering-in-place regulations, and social distancing practices have been successful in controlling the spread of the coronavirus, and fewer people are becoming infected with COVID-19 and dying from it.  This success is showing that if we work together, we can keep each other and our communities safe and healthy.  Because of this, several state and city governments are starting to ease or remove these strict measures, and to let people go back to work. 

However, there is a risk that the COVID-19 pandemic could get worse again, and cause more suffering and death.  Because there still is no vaccine or cure, COVID-19 remains a serious threat that can reappear at any time or place.  It can begin spreading again if we stop being careful.  Easing lock-downs, opening up businesses and schools, and relaxing social distancing orders could allow the COVID-19 pandemic to get out of control once again, and put people’s lives at risk.

For now, lock-downs and strict social distancing measures remain in place in many US states.  Many experts believe that several things are necessary before lock-downs and strict social distancing measures are loosened, including the capability to test many more people for the coronavirus, to isolate people who are infected, and to identify and monitor their close contacts.  Many other government officials believe it is safe to begin loosening lock-downs and strict social distancing measures right now.  Some state and local governments in the US have therefore started loosening these measures, or are at least considering loosening doing so.  Regardless of what course we choose, however, if we continue to care for each other and our communities, we will control the COVID-19 pandemic.  We’re all in this together.

**Intentions for COVID-19 risk-reducing behaviors**

**How fully do you plan to follow each of these guidelines over the next month?**

| 0 | 10 | 20 | 30 | 40 | 50 | 60 | 70 | 80 | 90 | 100 |
| --- | --- | --- | --- | --- | --- | --- | --- | --- | --- | --- |

**Please use the slider bar to indicate your answer, using the response scale below:
0 = I am NOT planning to follow this guideline AT ALL
100 = I AM planning to follow this guideline FULLY**

| Avoid gatherings of 10 or more people. | 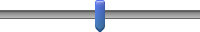 |
| --- | --- |
| If someone in your house has tested positive, keep the entire household at home. Do not go to work or school. | 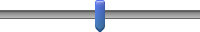 |
| Avoid optional travel. | 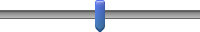 |
| Avoid optional shopping trips. | 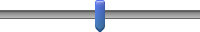 |
| Avoid optional social visits. | 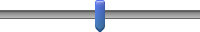 |
| Avoid eating or drinking in restaurants, bars, & food courts. Use drive-though, pickup, and delivery options. | 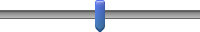 |
| Do not visit nursing homes or retirement or long-term care facilities unless to provide critical assistance. | 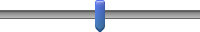 |
| Practice good hygiene, such as washing your hands, especially after touching frequently used items or surfaces. Avoid touching your face. | 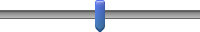 |
| Keeping a safe distance of at least 6 feet from other people. | 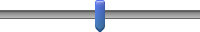 |
| Wear a mask covering your nose and mouth. | 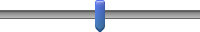 |
| Self-isolating if you have symptoms of COVID-19. | 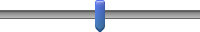 |
| Self-quarantining if you have been exposed to someone with a known COVID-19 infection, or have been traveling to another country or area identified as a COVID-19 hot spot. | 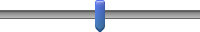 |
| Calling your health care provider if you develop symptoms including cough, fever, or difficulty breathing. | 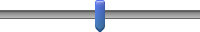 |
| Practice strict sheltering-in-place (staying at home, away from work and school) | 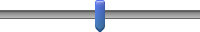 |
